# Identification of metabolite extraction method for targeted exploration of antimicrobial resistance associated metabolites of *Klebsiella pneumoniae*

**DOI:** 10.1038/s41598-022-12153-0

**Published:** 2022-05-27

**Authors:** Ashok Kumar, Sevaram Singh, Sonu Kumar Gupta, Shailesh Kumar, Shrikant Kumar, Rita Singh, Lovnish Thakur, Manoj Kumar, Arti Kapil, Yashwant Kumar, Niraj Kumar

**Affiliations:** 1 Translational Health Science and Technology Institute, NCR Biotech Science Cluster, 3rd Milestone, Faridabad – Gurugram Expressway, PO box #04, Faridabad, 121001 India; 2 Jawaharlal Nehru University, New Mehrauli Road, Delhi, 110067 India; 3 Department of Microbiology, All India Institute of Medical Sciences, New Delhi, 110029 India

**Keywords:** Biochemistry, Biological techniques, Biotechnology, Microbiology

## Abstract

Antimicrobial resistant *Klebsiella* *pneumoniae* (*K. pneumoniae*), as being a pathogen of critical clinical concern, urgently demands effective therapeutic options. However, the discovery of novel antibiotics over the last three decades has declined drastically and necessitates exploring novel strategies. Metabolomic modulation has been the promising approach for the development of effective therapeutics to deal with AMR; however, only limited efforts have been made to-date, possibly due to the unavailability of suitable metabolites extraction protocols. Therefore, in order to establish a detailed metabolome of *K. pneumoniae* and identify a method for targeted exploration of metabolites that are involved in the regulation of AMR associated processes, metabolites were extracted using multiple methods of metabolites extraction (freeze–thaw cycle (FTC) and sonication cycle (SC) method alone or in combination (FTC followed by SC; FTC + SC)) from *K. pneumoniae* cells and then identified using an orbitrap mass analyzer (ESI-LC–MS/MS). A total of 151 metabolites were identified by using FTC, 132 metabolites by using FTC+SC, 103 metabolites by using SC and 69 metabolites common among all the methods used which altogether enabled the identification of 199 unique metabolites. Of these 199, 70 metabolites were known to have an association with AMR phenotype and among these, the FTC + SC method yielded better (identified 55 metabolites), quantitatively and qualitatively compared to FTC and SC alone (identified 51 and 41 metabolites respectively). Each method of metabolite extraction showed a definite degree of biasness and specificity towards chemical classes of metabolites and jointly contributed to the development of a detailed metabolome of the pathogen. FTC method was observed to give higher metabolomic coverage as compared to SC alone and FTC + SC. However, FTC + SC resulted in the identification of a higher number of AMR associated metabolites of *K. pneumoniae* compared to FTC and SC alone.

## Introduction

Antimicrobial Resistance (AMR) has become one of the major escalating threats to global public health^1^. It underlines the urgent need for action as resistant infections not only require expensive treatments, prolonged hospital stays, and increased healthcare costs but also loss of man-hour productivity^2^. Currently, ~ 0.7 million people die globally each year of drug resistance illnesses due to bacterial infections and this is estimated to increase to ~ 10 million deaths per year by 2050 including 4.1 million deaths from Africa and 4.7 million from Asia, and cost $100 trillion for their treatment and infrastructure development^3^. Therefore, understanding the mechanisms regulating the emergence (and/or spread) of AMR among bacterial pathogenesis may be of critical importance.

*Klebsiella pneumoniae*, a gram-negative γ-proteobacteria belonging to the family *enterobacteriacae*, is well known to cause various life-threatening infections, including hospital-acquired infections (HAIs; such as pneumonia, bloodstream infections (BSIs), urinary tract infection (UTIs)), and community-acquired infections (CAIs; such as necrotizing pneumonia, pyogenic liver abscesses and endogenous endophthalmitis)^4^. *K. pneumoniae*, along with severe pathogenesis and high prevalence, is well known to show multi/extensively-drug resistance (MDR/XDR) phenotype to even next-generation antibiotics (such as cephalosporins, aminoglycosides, fluoroquinolones, and carbapenems) and hence has only limited therapeutic options for treating the MDR/XDR infected *K. pneumoniae* patients^5^. Therefore, the World Health Organization (WHO) has recently declared extended-spectrum β-lactamases (ESBLs) and carbapenem-resistant *K. pneumoniae* (CRKP) as a critical pathogen to public health^6^. In Europe alone, ESBL and CRKP strains reportedly account for > 90,000 infections and > 7000 deaths annually^7^. Moreover, the pathogen has been considered as a major source and shuttle for antibiotic resistance-associated genes among the bacterial pathogen for the emergence and spread of AMR^8^. However, despite such a clinical relevance*,* our understanding about the mechanism(s) of emergence and spread of AMR in the *K. pneumoniae* pathogen is limited. So far, a number of targeted attempts using molecular genetics, transcriptomics and proteomics-based tools have been made, but the gradually decreasing trend in the discovery of novel antibiotics highlights the urgency for exploring novel strategies to deal with the upcoming problem of AMR.

Recently, investigations of metabolomic modulation among pathogens are one of the promising ways to deal with the problem of AMR. For example, conjugation of aminoglycosides with fructose and fumarate was shown to increase the activity of aminoglycosides against *S. aureus* and *E. coli*^9^. Similarly, conjugation of aminoglycosides with glyoxylate was reported to decrease the activity of aminoglycosides against the *P. aeruginosa*^10^. Moreover, a significant inhibition of the citrate cycle, pentose phosphate pathway, amino acid and nucleotide metabolism were observed during treating the multidrug-resistant *K. pneumoniae* by a bacteriophage-polymyxin combination highlighting the potential targets and pathways for improving the efficacy of existing antimicrobials or for novel antimicrobial development^11^. However as of now, only limited such attempts for investigating metabolome of *K. pneumoniae* have been made, primarily using Gas chromatography–mass spectrometry (GC–MS) and Nuclear Magnetic Resonance (NMR) techniques which are known to have low sensitivity and the metabolome coverage^12–15^. Hence, the metabolome of the pathogen has largely remained yet unknown and the unavailability of the appropriate protocols for the purpose may potentially be one among the reasons*.*

Therefore, in this investigation, we aimed to establish the comprehensive intrinsic metabolomic profile of *K. pneumoniae* and identify the method for targeted investigation of AMR associated metabolites. Freeze–thaw cycle (FTC) and sonication cycle (SC) are the most commonly used approaches for metabolite extraction and are known to have specificity and biasness toward specific metabolites^16^. We have utilized both (FTC and SC) along with their combination (FTC + SC) to increase the metabolome coverage. The extracted metabolites were identified and investigated using an orbitrap fusion tribrid mass analyzer (Thermo-Scientific, ESI-LC–MS/MS) as it may potentially help in further increasing the metabolome coverage.

## Materials and methods

### Bacterial cell culture

*K. pneumoniae* strain (ATCC 33495; ampicillin resistant) was precultured aerobically in MHB (Difco™), for ~ 12 h at 37 °C with 220 rpm. The fresh cultures were then inoculated (1: 500 v/v) in fresh MHB and incubated under aerobic conditions at 37 °C until the mid-exponential phase of growth (~ 4 h post-inoculation). At the time of sample collection, cultures equivalent to 10^7^ cells were centrifuged for 10 min at 4 °C and 8000 g, the supernatant was discarded and the cell pellets were stored at − 80 °C till further analysis.

### Metabolite extraction

**Figure 1:**
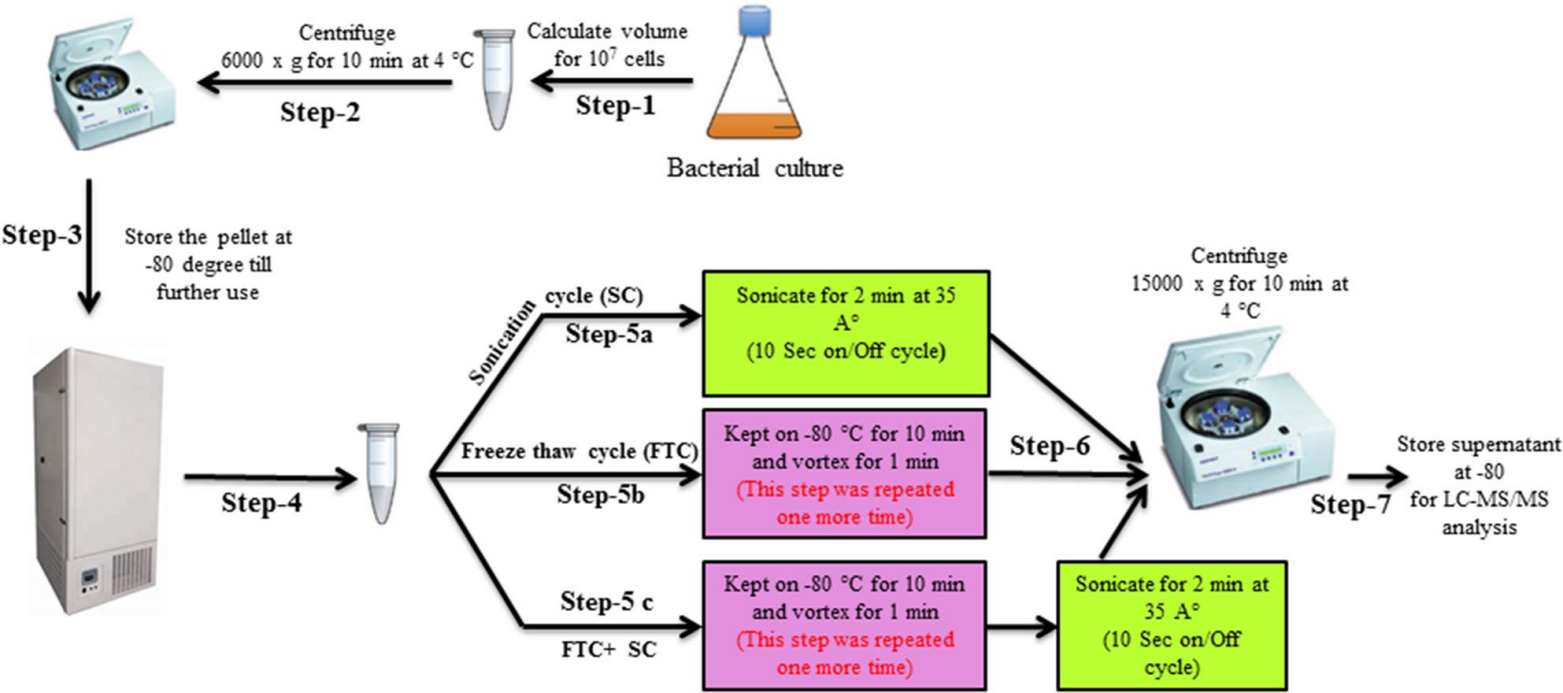
Workflow of metabolite extraction: Cell pellets were used for metabolites extraction using 3 extraction protocols (i) Sonication cycle, SC (ii) Freeze–thaw cycle, FTC (iii) Freeze–thaw followed sonication cycle, FTC + SC.

Metabolites were extracted from the quenched cells using 100% methanol (~ 500 μl). Varying concentration (10 ng/ml, 20 ng/ml 40 ng/ml, 80 ng/ml, 160 ng/ml) of 13C-labeled l-valine, an internal standard, was also added in each sample to enable plotting of the standard curve with its recovery data and evaluating the metabolites extraction efficiency of each method. This was also expected to contribute in ensuring that the differences observed in metabolomic profiles are reproducible (at varying concentration of the internal standard) and potentially true findings (not due to steps associated with a particular method). For the sonication cycle, the cells pellet frozen at − 80 °C was thawed for 10 min and resuspended in 500 μl of 100% chilled methanol (MS-grade, Sigma) and sonicate for 2 min at 35 A° for 10-s on-and-off cycles (Fig. 1 step 5a). For better coverage of different classes of metabolite extraction, we performed a free-thaw cycle, after resuspending the frozen cell pellet, freeze–thaw cycles were repeated thrice (Fig. 1 step 5b). Similarly, for FTC + SC, after resuspending the frozen cell pellet, the freeze–thaw cycle is repeated thrice and followed by sonication as described earlier (Fig. 1 step 5c). For the collection of metabolites, cells were pelleted down by centrifugation at 15,000×*g* for 10 min at 4 °C and the supernatant (~ 400 μl) was collected after centrifugation in a separate pre-labelled microfuge tube without disturbing the pellet. A fraction of each sample (100 μl) was dried using a speed vacuum. Samples were stored at − 80 °C till further analysis.

### Separation and measurements of metabolites using ESI-LC–MS/MS

Each metabolite sample was resuspended in 25 μl of the methanol–water mixture (3:17, methanol: water), vortexed briefly, and centrifuged at 11000 rpm for 10 min at 4 °C. The metabolites were then separated on UPLC ultimate 3000 using a C18 column (HSS T3) and HILIC column (XBridge BEH Amide) maintained at 40 °C and 35 °C temperature respectively. For the reverse phase, the mobile phase was water + 0.1% formic acid (mobile phase A) and methanol + 0.1% formic acid (mobile phase B). For HILIC chromatography, mobile phase A was 20 mM ammonium acetate in the water of pH 9.0 and mobile phase B was 100% acetonitrile. The elution gradient for the reverse-phase started with 1% mobile phase B to 99% mobile phase B over 14 min. Similarly, the elution gradient for HILIC chromatography was 85% B to 10% B over 16 min. The flow rate of 0.3 ml/min for the reverse phase and 0.35 ml/min for HILIC chromatography respectively. The sample injection volume was 5 µl. The mass-spectrometric data was acquired in positive and negative mode at 120,000 resolutions in MS1 mode and 30,000 resolutions in data-dependent MS2 scan mode using on the Orbitrap fusion tribrid mass spectrometer (Thermo- Scientific) equipped with heated electrospray ionization (HESI) source using the previously described method^17^. We used spray voltage of 4000 and 35,000 V for positive and negative modes respectively. Sheath gas and auxiliary gas were set to 42 and 11 respectively. Mass range was 60–900 m/z, AGC (Automatic gain control) target at 100,000 ions and maximum injection time was 50 ms for MS and AGC target were 20,000 ions and maximum injection time 60 ms for MS/MS was used.

### Data processing

All acquired data was processed using the Progenesis QI for metabolomics (Water Corporation) software using the default settings. The untargeted workflow of Progenesis QI was used to perform retention time alignment, feature detection, deconvolution, and elemental composition prediction. Metascope plug of Progenesis QI has been used for the in-house library of 950 metabolites with accurate mass, fragmentation pattern and retention time for database search. We have also used the online available spectral library for further confirmation of identification. Any peak that was detected in all 36 replicates of any metabolite extraction method and receives an identification using an in-house library (retention time error up to ± 1 min, accurate mass error up to 5 ppm and for MS/MS it was up to 20 ppm and fragmentation pattern match) and spectral library match was considered as identified metabolites.

### Statistical analysis

For each of the metabolite extraction protocols (SC, FTC and FTC + SC), 36 independent experiments were performed. Processing of the raw data leads to the identification of a total of 103, 151, and 132 metabolites using the SC, FTC and FTC + SC methods, respectively. All the further statistical and functional analysis including PCA, heat map, molecular pathways identification, and analysis of variance (ANOVA) was done based on the identified peak intensity using the freely available online software MetaboAnalyst 5.0. For analysis of PCA, heat maps and ANOVA data was put in a matrix, with samples in rows and features in columns. Before the final analysis of data, an integrity check was performed, and raw data were normalized using sum methods followed by Pareto scaling (Mean-centered and divided by the square root of the standard deviation of each variable). The differences among different metabolite extraction methods were analyzed using the average peak intensity of all replicates (n = 36) for each metabolite extraction method (SC, FTC and FTC + SC).

## Results

### Identification of metabolites

**Figure 2:**
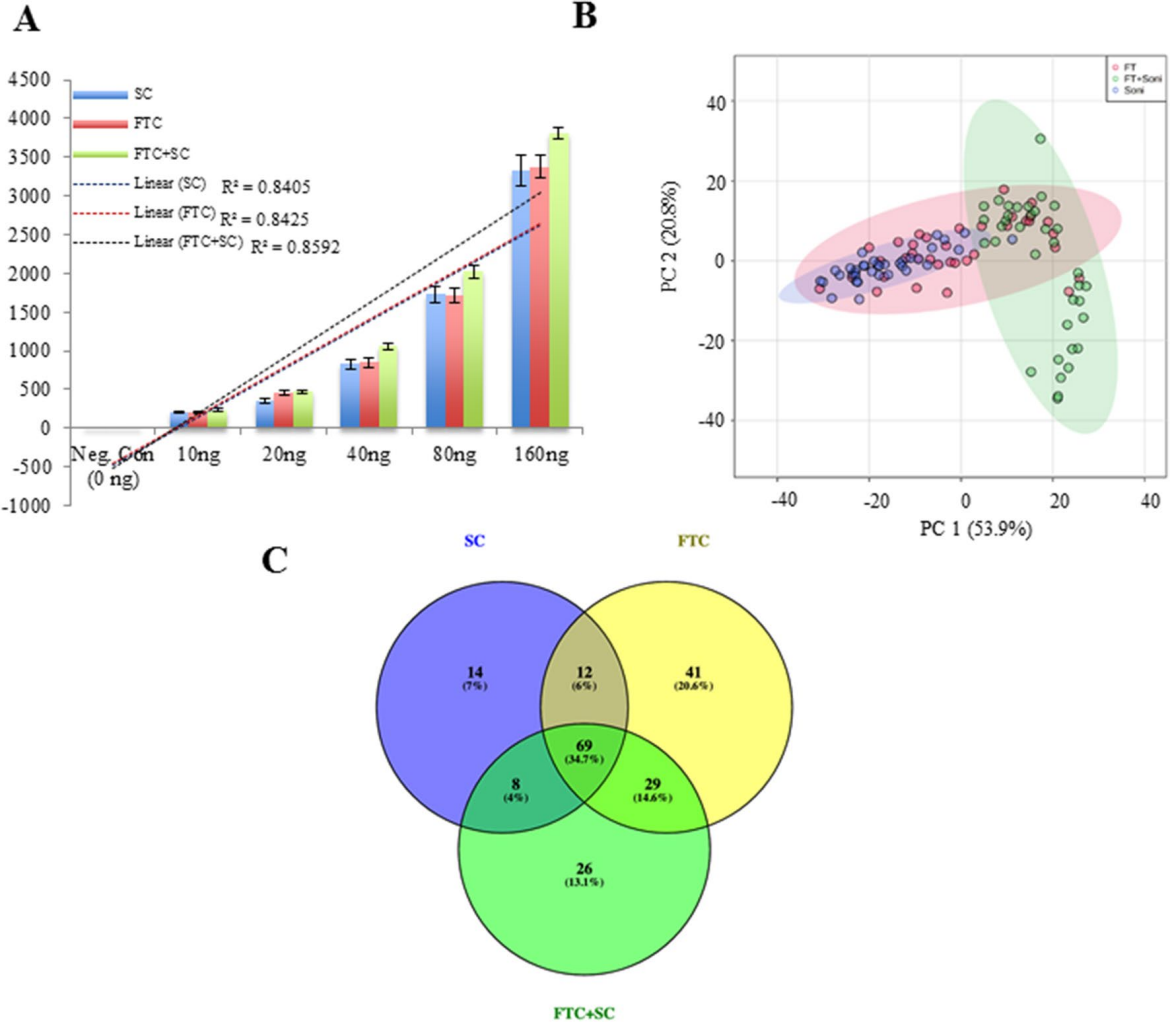
Analysis of sample processing process efficiency and identified metabolites: (**A**) Parity plot for ^13^C-labeled l-valine for comparison of metabolites extraction efficiency of (i) SC, (ii) FTC, (iii) FTC + SC. (**B**) PCA analysis (**C**) Venn diagram illustrating the overlapping and SC, FTC and FTC + SC metabolite extraction protocol-specific differential metabolites. The data included information generated using 36 independent replicate samples for each metabolite extraction method.

*K. pneumoniae* cell pellets collected from exponentially growing cultures were spiked with the various concentrations of ^13^C-labeled l-valine (10 ng/ml, 20 ng/ml 40 ng/ml, 80 ng/ml, 160 ng/ml) as internal standard. The metabolites were then extracted using freeze–thaw cycle (FTC), sonication cycle (SC), and their combination (FTC + SC) to increase metabolome coverage and to address the question of the method-specific effect on metabolomic profile (Fig. 1). The extracted metabolites were then separated using UPLC ultimate 3000 with C18 (non-polar) and HILIC column (polar), detected and quantitated using the most sensitive equipment for metabolite detection, Orbitrap fusion Mass spectrometer equipped with heated electrospray ionization (HESI). The analysis was performed using Progenesis QI empowered with the in-house developed and validated metabolite library for their accurate mass, fragmentation pattern and retention time. At first, quantitative data for recovery of ^13^C-labeled l-valine (internal standard) from each method was analyzed using a parity plot for comparing the extraction efficacy of each metabolites extraction method and to ensure that the observed method-specific effect on the metabolomic profile is a true finding and not because of the procedure/steps associated with extraction methods. Although the concentration of the internal standard (^13^C-labeled l-valine) was observed to be comparable in metabolite samples extracted using the freeze–thaw cycle (R^2^ = 0.8425) or sonication (R^2^ = 0.8405), but it was slightly higher in metabolite samples extracted using a combination of freeze–thaw cycle and sonication process (FTC + SC, R^2^ = 0.8592) compared to any of the individual methods alone (Fig. 2A). The data were also analyzed using PCA analysis to identify commonness and uniqueness among the metabolomic profile generated using the three extraction protocols, FTC, SC, and FTC + SC. The data showed a noticeable degree of overlaps between FTC, SC, and FTC + SC along with an obvious degree of separation between FTC, SC and FTC + SC (Fig. 2B). This variation reflects quantitative as well as a qualitative change in the metabolomic profile acquired using FTC, SC and FTC + SC.

A total of 151 metabolites were identified by using FTC method whereas, 103 by SC and 132 by FTC + SC. Among these, 69 metabolites were common among all the extraction protocols used (FTC, SC & FTC + SC), 12 were common between SC and FTC, 8 between the SC and FTC + SC and 29 between FTC and FTC + SC (Table 1 and Fig. 2C). Of the identified metabolites, 81 (~ 41%) showed protocol-specific specificity and biasness (Table 1 & Supplementary Table 1). A total of 41 metabolites (~ 20.6%) were uniquely identified in metabolite samples extracted using FTC and 14 metabolites (~ 7%) were uniquely identified in metabolite samples extracted using the sonication method, whereas 26 metabolites (~ 13.1%) were uniquely identified in metabolite samples extracted using FTC + SC (Table 1).

**Table 1:**
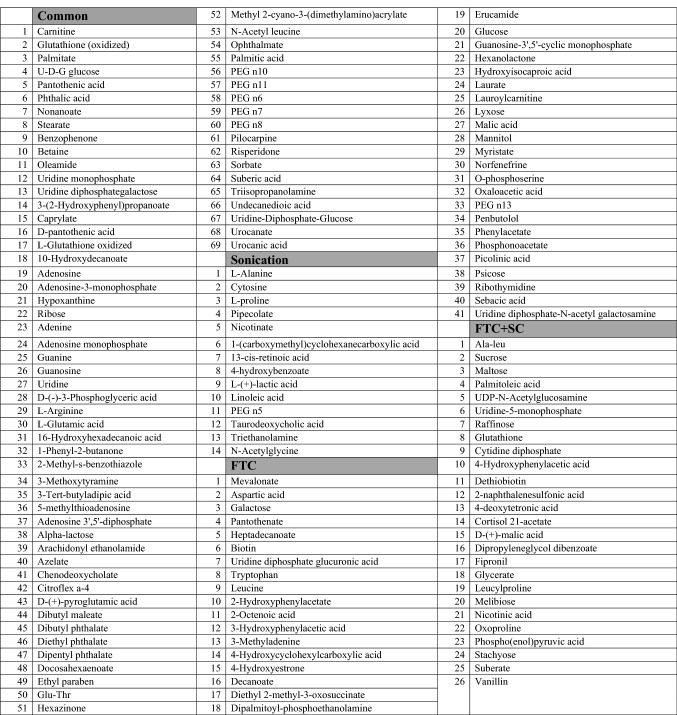
List of extraction protocol specific unique metabolites identified using SC, FTC and FTC + SC along with common metabolites (identified by all methods).

### Biochemical categorization of identified metabolites

**Figure 3:**
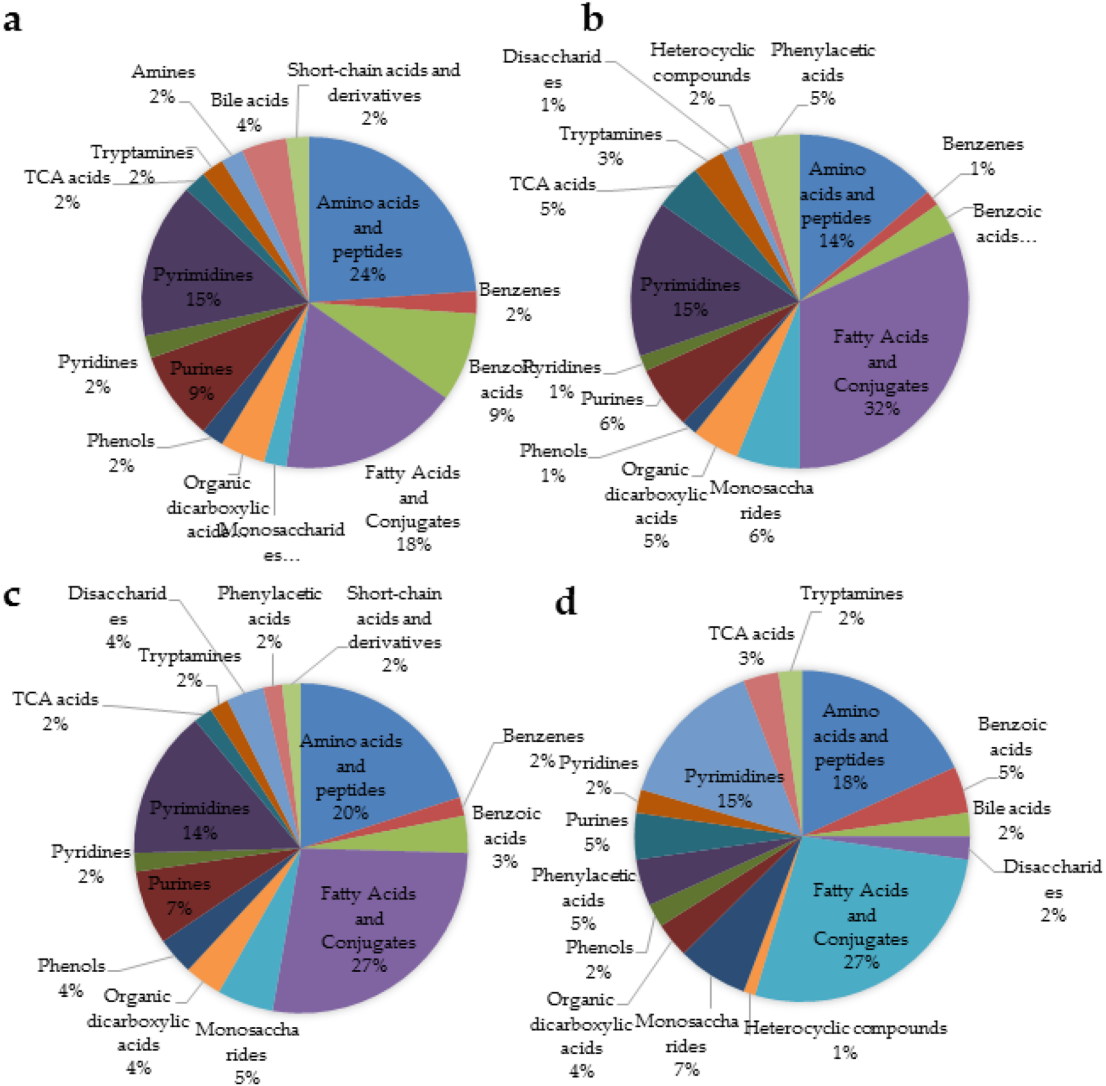
Pie-chart displaying the chemical specificity and biasness of metabolites extraction protocol on metabolomic profile (**a**) Sonication cycle, (**b**) Freeze–thaw cycle, (**c**) FTC + SC, (**d**) Chemical classification of all metabolites irrespective of the extraction method.

Metabolite extraction methods were observed to show biasness towards specific chemical classes/subclass of metabolites. The freeze–thaw method of metabolite extraction was observed to be more biased towards fatty acids and conjugates (32%) followed by pyrimidines (15%) and amino acids and peptides (14%) with exclusivity heterocyclic compounds (2%) (Fig. 3b). Similarly, the sonication method was observed to be more biased towards the amino acids and peptides (24%), followed by fatty acids and conjugates (18%) and pyrimidines (15%) with the exclusivity for amines (25%), in metabolite samples extracted using SC method (Fig. 3a). Whereas, utilization of a combination of both approaches (FTC + SC) was observed to be biased towards fatty acids and conjugates (27%), followed by amino acids and peptides (20%) and pyrimidines (14%) (Fig. 3c). Of the overall identified metabolites (199), approximately 30% of metabolites fell in the category of fatty acids and conjugants. Of the rest, 20% were represented by amino acids and peptides, 16% by pyrimidines, 7% by monosaccharides and 5% by purines (Table 2, Supplementary Table 1 and Fig. 3d).

**Table 2:** List of AMR or it's associated identified metabolites with peak intensity.

S.no.	Metabolite	Method of detection (peak intensity)		
		SC	FTC	FTC + SC
**Cell wall biosynthesis**				
1	Carnitine	413.60	210.37	234.03
2	Glutathione (oxidized)	2261.78	3313.24	2277.07
3	Palmitate	328,732.17	175,542.63	32,988.38
4	U-D-G glucose	12,507.41	7548.63	9292.17
5	Pantothenic acid	435.51	486.74	634.83
6	Phthalic acid	1699.60	1608.78	2197.18
7	Nonanoate	1126.71	1016.35	759.79
8	Stearate	100,943.83	99,892.75	83,283.90
9	Benzophenone	1243.86	1301.05	1341.30
10	Betaine	18,091.82	16,474.18	14,153.99
11	Oleamide	4671.00	4992.14	4529.73
12	Uridine monophosphate	720.08	415.26	713.81
13	Uridine diphosphategalactose	338.61	494.66	591.62
14	3-(2-Hydroxyphenyl)propanoate	449.12	366.04	379.49
15	Octadecanedioic acid	–	554.22	1721.41
16	Trehalose	–	1101.25	637.29
17	l-Aspartate	–	265.92	617.25
18	l-lysine	–	213.47	306.04
19	l-Alanine	609.26	–	–
20	Mevalonate	–	57.02	–
21	Aspartic acid	–	567.81	–
22	Galactose	–	300.01	–
23	Pantothenate	–	278.07	–
24	Ala-leu	–	–	1082.98
25	Sucrose	–	–	10,280.49
26	Maltose	–	–	1286.16
27	Palmitoleic acid	–	–	2738.64
28	UDP-N-Acetylglucosamine	–	–	1655.90
29	Uridine-5-monophosphate	–	–	3243.20
30	Raffinose	–	–	439.59
Total		15.00	22.00	25.00
**Cell membrane**				
31	Caprylate	551.49	548.69	699.02
32	d-pantothenic acid	658.56	702.47	776.97
33	l-Glutathione oxidized	7492.62	8310.20	5288.27
34	10-Hydroxydecanoate	539.80	825.35	654.67
35	Glycolic acid	437.88	−	540.63
36	Hexadecanedioic acid	−	226.35	386.67
37	Heptadecanoate	−	136.80	−
38	Biotin	−	1307.61	−
39	Uridine diphosphate glucuronic acid	−	2243.12	−
40	Glutathione	−	−	1107.25
Total		5.00	8.00	7.00
**Nucleotide metabolism**				
41	Adenosine	4086.96	4782.98	5018.04
42	Adenosine-3-monophosphate	483.44	382.52	1058.15
43	Hypoxanthine	3212.21	944.57	571.10
44	Ribose	644.30	643.42	559.87
45	Adenine	387.66	214.02	367.07
46	Adenosine monophosphate	1342.57	1141.64	2540.13
47	Guanine	3449.86	7865.84	3635.78
48	Guanosine	543.53	349.92	1084.45
49	Uracil	941.17	1179.20	−
50	4-Aminobenzoic acid	393.54	−	455.21
51	Inosine	−	363.91	1702.13
52	Ump	−	214.79	503.96
53	Cytidine monophosphate	−	283.23	640.49
54	Cytosine	508.32	−	−
55	Uridine	343.83	427.97	843.25
56	Cytidine diphosphate	−	−	379.63
Total		13.00	14.00	14.00
**Protein synthesis**				
57	d-(-)-3-Phosphoglyceric acid	854.48	521.40	980.56
58	l-Arginine	378.60	339.70	427.68
59	l-Glutamic acid	4963.65	3184.31	1901.57
60	Alloisoleucine	988.76	−	794.83
61	Isoleucine	2618.71	−	2912.32
62	NAD	−	15,879.67	3470.52
63	Glutamic acid	−	1594.75	197.33
64	l-Proline	3921.73	−	−
65	Pipecolate	479.67	−	−
66	Nicotinate	475.13	−	−
67	Tryptophan	−	463.37	−
68	Leucine	−	2952.60	−
69	4-Hydroxyphenylacetic acid	−	−	391.96
70	Dethiobiotin	−	−	331.84
Total		8.00	7.00	9.00

## Discussion

Antimicrobial resistant, *K. pneumoniae* infection, because of the unavailability of effective antimicrobials, has been of great clinical concern worldwide^18^. Besides enormous efforts, hardly any antibiotic has been discovered over the last few decades, and this necessitates exploring novel strategies to deal with the emerging problem of AMR^19^. Recently, modulation of bacterial metabolomic flux has been shown to potentiate the efficacy of antibiotics uncovering the potential of metabolomics to deal with the problem of AMR. However, only a few efforts have been made in this direction which may potentially be due to the unavailability of targeted methods for the purpose. Therefore, in this investigation, besides developing the comprehensive intrinsic metabolomic profile of *K. pneumoniae*, we have tried to identify a potential method of metabolite extraction for the targeted investigation of AMR associated metabolites. Since the intrinsic biochemical composition and cellular architecture among most of the *K. pneumoniae* strains is highly expected to be similar as they all belong to a common species^14^, a single strain of *K. pneumoniae* (ATCC 33495) was utilized in this study.

### Analysis of identified metabolites and metabolite extraction method

The metabolites were extracted using the two most commonly used methods, free–thaw cycle (FTC) and sonication cycle (SC) alone as well as in combination (FTC followed by SC, FTC + SC). The highest number of metabolites were identified using FTC followed by FTC + SC and SC, indicating its suitability for achieving higher metabolome coverage (Table 1). To date, only a few ‘OMICS’ based studies targeting compressive metabolomic analysis of *K. pneumoniae* have been performed^11–15,20,21^. Recently, in a GC–MS based study, 36 K*. pneumoniae* specific metabolites were identified, of which, 8 metabolites (25%) were identified in our study also^16^. In contrast, none of the volatile metabolites, identified in two other studies using GC–MS, was detected in our study which may be possible because GC–MS is known to enrich and capture volatile metabolites whereas, LC–MS is for soluble metabolites^12,14^. However, ~ 43% of metabolites identified in previous studies using NMR were also detected in our study^14,15^. Although the reason for this low overlap remains unexplainable, it may be speculated that the NMR is known to have a low sensitivity and is reported to be incomparable with LC–MS/MS. Taken together, this data also suggests that the use of all three metabolite identification approaches has a potential to further increase the metabolomic coverage of the pathogen under investigation. Moreover, this higher number of identified metabolites in our study (199) compared to previously published studies may be potential because (1) we have used three extraction methods, and/or (2) ESI-LC–MS/MS is known to have wide coverage and higher sensitivity compared to GC–MS and NMR.

**Figure 4:**
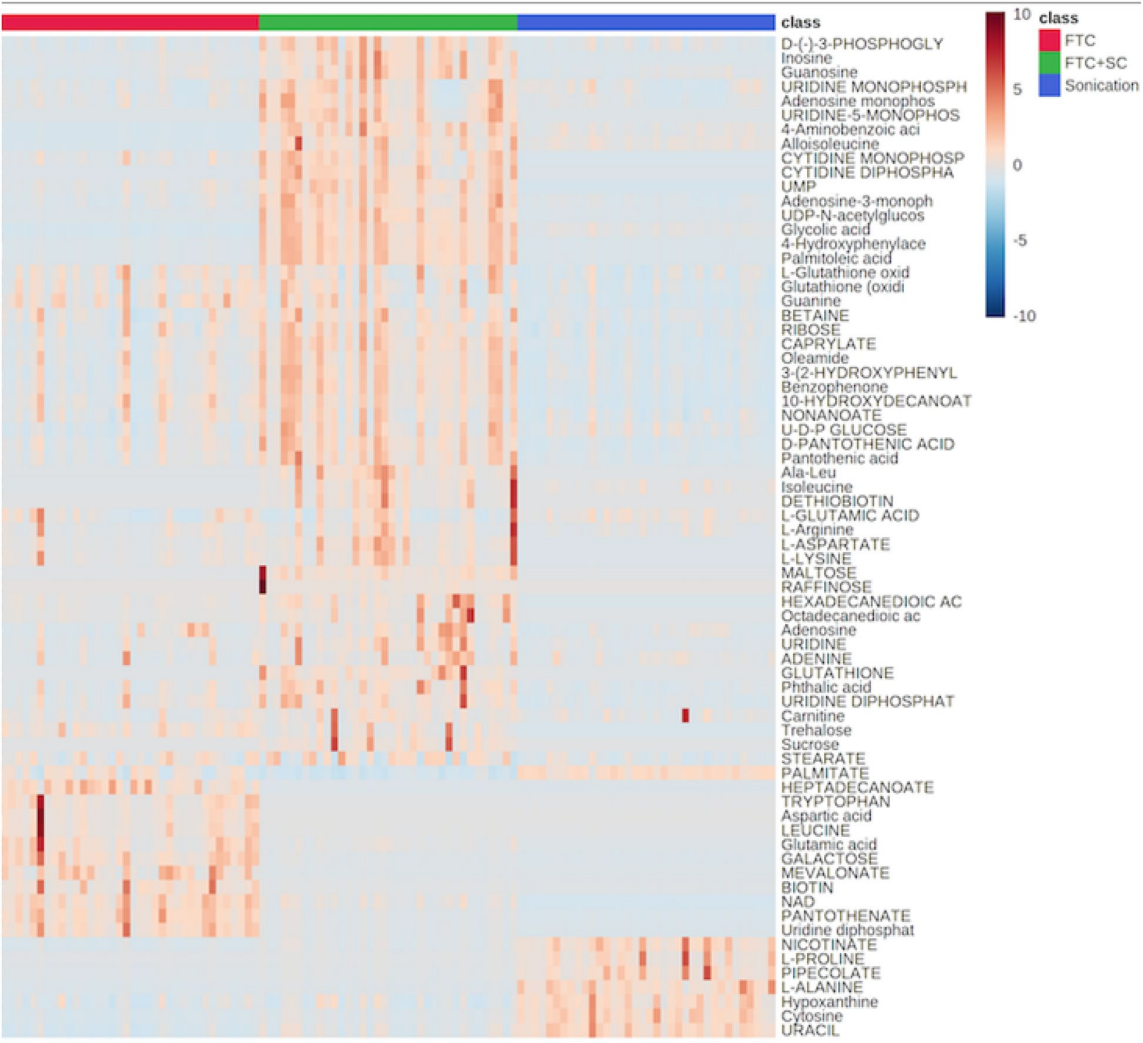
Heat map displaying qualitative enrichment occurred in the AMR associated metabolites. *Red* FTC, *Green* FTC + SC, *Blue* Sonication cycle.

The FTC was found to identify ~ 32.24% more metabolites than SC and 13.16% more than FTC + SC indicating its superiority over other methods (Table 1, Fig. 4 and Supplementary Fig. 1). FTC + SC was expected to enable the identification of the highest number of metabolites as FTC alone facilitates the identification of overall 151 metabolites and SC alone was observed to enable specific identification of 14 metabolites; which indicates the potential degradation of a few metabolites during sonication. Whereas, the intensity of identified metabolites was observed to be higher in FTC + SC compared to FTC and SC indicating its utility in quantitative and targeted metabolomics of the pathogen, *K. pneumoniae* (Supplementary Fig. 1). Moreover, each extraction method showed an obvious degree of similarity and biasness/specificity towards a particular class/subclass of metabolites (Fig. 3). As in the case of SC, the most abundant class of metabolites was found to be amino acids and peptides (24%, Fig. 3a) but in FTC, it was fatty acids and conjugates (32%, Fig. 3b). The results were found to be somewhat intermediate in FTC + SC, 20% for amino acids and peptides and 27% for fatty acids and conjugates (Fig. 3c). In terms of qualitative coverage, FTC + SC was found to be most efficient over FTC and SC for metabolites extraction (Supplementary Fig. 1).

### Analysis of AMR associated metabolites

It is known that all classes of antibiotics, except a few, target anyone from the four major cellular processes; (1) cell wall biosynthesis, (2) cell membrane permeability, (3) DNA replication and (4) Protein biosynthesis^22^. Efficient detection of metabolites associated with these processes may help to understand the molecular mechanism of action of antibiotics and their effectiveness (increased/reduced activity). Therefore, the role of each identified metabolite in cell wall biosynthesis, cell permeability, DNA replication, protein biosynthesis and other cellular processes was extensively searched in the published literature to explore their association with AMR associated process (cell proliferation and/or cell death). FTC + SC method was observed to identify highest number of AMR associated metabolites (55 metabolites) compared to FTC (51 metabolites) or SC (41 metabolites) alone indicating its suitability over other methods for investigation of AMR associated metabolites (Table 2). However, metabolite-specificity of the methods was also observed indicating utility of other methods for targeted exploration of metabolites. The potential association of a few identified metabolites with respect to AMR associated cellular process may be as detailed below.

#### Cell wall biosynthesis

Bacterial cell wall biosynthesis is one of the major targets of β-lactam, cephalosporins, carbapenem and monobactams. Of these, β-lactam are the most frequently used cell wall biosynthesis inhibitor against both Gram-positive and Gram-negative bacterial infection^23^. The identified metabolites included a number of peptidoglycan biosynthesis precursors such as UDP-*N*-acetylglucosamine (UDP-GlcNAc), UDP-glucose, uridine monophosphate, uridine di-phosphate galactose, uridine diphosphate glucuronic acid, galactose, pantothenic acid, stearate, betaine, trehalose, l-aspartate, palmitoleic acid, l-alanine, l-lysine, mevalonate, aspartic acid, alanine-leucine and palmitoleic acid. Peptidoglycan is the major constituent of the bacterial cell wall, which is a heteropolymer consisting of *N*-acetylglucosamine (NAG) and *N*-acetyl-muramic acid (NAM) bridged by β-1,4 glycosidic bond^24,25^. UDP-GlcNAc is synthesized from fructose-6-phosphate (glycolysis intermediates) in a four-step process catalyzed by GlmS, GlmM, and GlmU^26^. It is also an important molecule for the biosynthesis of various macromolecules such as teichoic acid in Gram-positive bacteria) and lipopolysaccharides (LPS) in Gram-negative bacteria^27^. UDP-glucose is also an essential metabolite involved in the synthesis of trehalose that is involved in the biosynthesis of bacterial envelopes particularly LPS and the capsule. LPS and capsules constitute the key pathogenicity determinant in *K. pneumoniae* bloodstream infection^28^. The identified l-lysine and d-Alanine are also essential components of the pentapeptide bridge (d-Ala-d-Ala moiety, a peptidoglycan precursor) that is required for peptidoglycan biosynthesis.

#### Bacterial cell membrane

Bacterial cell membrane is targeted by a number of synthetic antimicrobial peptides such as polymyxin-B & -E, daptomycin, telomycin and cinnamycin^29^. The outer monolayer of Gram-negative bacteria contains LPS as the major lipid constitute^30^. Polymyxin disrupts membrane integrity by displacing the cations (Ca^+2^/Mg^+2^) in outer membrane area, by associating with the negatively charged LPS and leading to cell lysis^31^. The identified metabolites were known to be involved in oxidative and osmotic stress regulation (such as GSH and hexadecanedioic acid) and a few were structural components of the cell membrane (such as 10-hydroxydecanoate, heptadecanoate and uridine diphosphate glucuronic acid). GSH is already known to play an important role in the maintenance of redox balance in the cytoplasm and defense against many toxic compounds, including heavy metals and antibiotics^32^. A biological level of 10 mM of GSH is reported in most Gram-negative bacteria. Whereas, in a study conducted using ESKAPE and Non-ESKAPE pathogens, 30 mM GSH concentration was found to disrupt the biofilm formation and decrease the pathogenic growth by more than 50%^33^. Therefore, it may be interesting to explore how *K. pneumoniae* regulates the GSH level during intervention with cell membrane-targeting antibiotics. Similarly, identified D-pantothenic acid (D-PA) which is a crucial water-soluble B-complex vitamin, the precursor of coenzyme A (CoA), and acyl carrier protein (ACP) is known to function in over 70 enzymatic pathways^34^. It is also known to help in cell membrane formation and deficiency of this metabolites cause osmotic lysis of cells. Hexadecanedioic acid & heptadecanoate are also found in the membrane and protect bacterial cells from osmotic lysis. The evaluation of these metabolites in response to bacterial treatment with cell membrane disrupting antibiotics may help to understand the molecular mechanism(s) of action of such antibiotics.

#### Protein biosynthesis inhibitors

Protein biosynthesis is also one of the major targets of antibiotics. Aminoglycosides, macrolides and lincosamides are some of the most frequently used protein synthesis inhibitors^35^. A number of metabolites, such as l-arginine, l-Glutamic acid, isoleucine, NAD, l-proline, and Tryptophan that are the important targets to evaluate the metabolomic perturbations induced by protein synthesis inhibitors such as chloramphenicol were identified (Table 2)^36^. Recently, the intervention of chloramphenicol has been shown to impinge on ammonia metabolism and induce an active response of arginine biosynthesis in *E. coli*^36^. Similarly using *Edwardsiella tarda,* it was demonstrated that the depressed concentration of alanine, aspartate and glucose was associated with the robust antimicrobial resistance phenotype. While the exogenous supplementation of alanine and glucose improved the uptake of the antibiotic and more easily cleared the pathogen load^37^. In this perspective, the identified alanine, glutamic acid, aspartic acid, and TCA metabolites such as oxaloacetic acid, citrate and malic acid are some of the crucial targets to potentiate the efficacy of aminoglycosides against the *K. pneumoniae*.

#### Bacterial nucleic acid synthesis inhibitors

Quinolones such as ciprofloxacin, ofloxacin, and nalidixic acid, etc. are the board spectrum antibiotics and are used against both Gram-positive and Gram-negative antibiotics^38^. They exert their action by disrupting the enzymatic activity of DNA gyrase, topoisomerase-II, and/or -IV and thus inhibiting bacterial nucleic acid synthesis^39^. The identified nucleotide metabolites [such as (adenosine, ribose, adenine, guanine, uridine, uracil, inosine, UMP and cytosine)] were found to be involved in a number of nucleotide biosynthesis or their degradation processes. In a study exploring the metabolomic perturbations induced by 9 antibiotics representing five mechanisms of action in *E. coli* have shown significant changes in overall intermediates of nucleotide and amino acid biosynthesis^36^. In this perspective identified metabolites such as uridine, uracil, 4-Aminobenzolic acid, inosine, UMP, octadecanedioic acid and the TCA intermediates may be of help in evaluating the metabolomic modulations induced by any antibiotics in *K. pneumoniae*. Which may help to increase the antimicrobial efficacy. Earlier, the conjugation of fluoroquinolones with l-serine was already reported to increase the activity of fluoroquinolones^40^.

#### Biofilm formation and development of persisters

It is known that bacterial pathogens utilize various strategies to survive against antibiotics, such as the acquisition of AMR genes, slowing down of central carbon metabolism, and formation of biofilm & persister cells^41^. Bacterial metabolism and metabolites are known to regulate the formation of the biofilm and development of persisters and hence are already known to contribute to the cost associated with treating resistant infections^42^. *K. pneumoniae* has been reported to grow biofilm on medical implants, catcher’s urinary tract and protect itself by biofilm formation indicating its pathogenic success as a urinary and respiratory pathogen^43^. However, little is known about how *K. pneumoniae* regulates metabolism during the formation of biofilm and the development of persistence. Recently, a metabolomic studies conducted on single- and dual-species biofilms of *Candida albicans* and *K. pneumoniae* have shown perturbation in central carbon, amino acid, vitamin, and secondary metabolisms (such as serine, leucine, arabitol, phosphate, vitamin B6, cyclo-(Phe-Pro), trehalose, and nicotinic acid) during biofilm formation^20^. The treatment of dual-species biofilm from *Candida albicans*/*K. pneumoniae* with antimicrobial peptides, WMR-K, was also shown to differentially regulate the expression of number of metabolites including amino acids, trehalose, pyruvic acid, glycerol and vitamin B6 metabolism^21^. Another metabolomic study conducted on *B. subtilis*, a well-known bacterial pathogen for studying the phenomenon of biofilm, showed significant dynamic changes in the primary metabolomic pathways, such as glycolysis, pentose phosphate pathway (PPP), tricarboxylic acid (TCA) cycle and nucleotide and amino acids biosynthesis pathways^44^. The level of TCA cycle intermediates (UDP-glucose, UDP-*N*-acetyl glucosamine and ADP-glucose) increased during the early-stage of biofilm formation, the level of branch chain/precursor amino acids (leucine/isoleucine and valine) peaked during the middle-stage of biofilm formation and the level of aromatic amino acids (such as phenylalanine, tyrosine and tryptophan) increased steadily over-time. In this way, the TCA intermediates (citrate, fumaric acid, oxaloacetic acids etc.), primary metabolism intermediates (isoleucine and tryptophan etc.) and *N*-acetylated amino acids and nucleotide sugar (*N*-acetyl-leucine, *N*-acetyl-tryptophan, *N*-acetyl glycine, UDP-Glucose, UDP-*N*-acetyl glucosamine) may be a good target to understand the biofilm formation and persistence phenomenon in *K. pneumoniae*. However, more studies need to be done to understand the *K. pneumoniae* metabolism during the formation of biofilm and the development of persistence.

## Conclusions

In summary, in this study, a comprehensive global metabolite profile of *K. pneumoniae* was established using 3 metabolites extraction protocols (i) sonication cycle (SC), (ii) freeze–thaw cycle (FTC), (iii) FTC followed by SC (FTC + SC). In terms of metabolomic coverage, FTC was found to be most efficient method of metabolite extraction for *K. pneumoniae* as it enables the identification of a highest number of metabolites (151) compared to SC (103) and FTC + SC (132). However, for targeting the AMR associated metabolites, FTC + SC was found to be the most efficient metabolites extraction protocol. As per our knowledge, this is the first study where such a comprehensive intrinsic metabolites profile *K. pneumonia* along with functional analysis of AMR associated metabolites; and the specificity and biasness of extraction protocols towards a particular class of metabolites is presented. The findings of this study may pave the way for exploring the emergence, spread, and detection of AMR along with targeted evaluation of perturbations in the metabolome of *K. pneumonia* and other gram-negative bacterial pathogens.

## Supplementary Information

Supplementary Information.

## Data Availability

The datasets used and/or analysed during the current study are available from the corresponding author on reasonable request.
