# Supplementary material for: Identification of metabolite extraction method for targeted exploration of antimicrobial resistance associated metabolites of Klebsiella pneumoniae
Source: Sci Rep. 2022 May 27;12:8939. doi: 10.1038/s41598-022-12153-0 (PMC9142494; doi:10.1038/s41598-022-12153-0)
Supplement: Supplementary file 1 — Supplementary Information. [file 41598_2022_12153_MOESM1_ESM.docx]

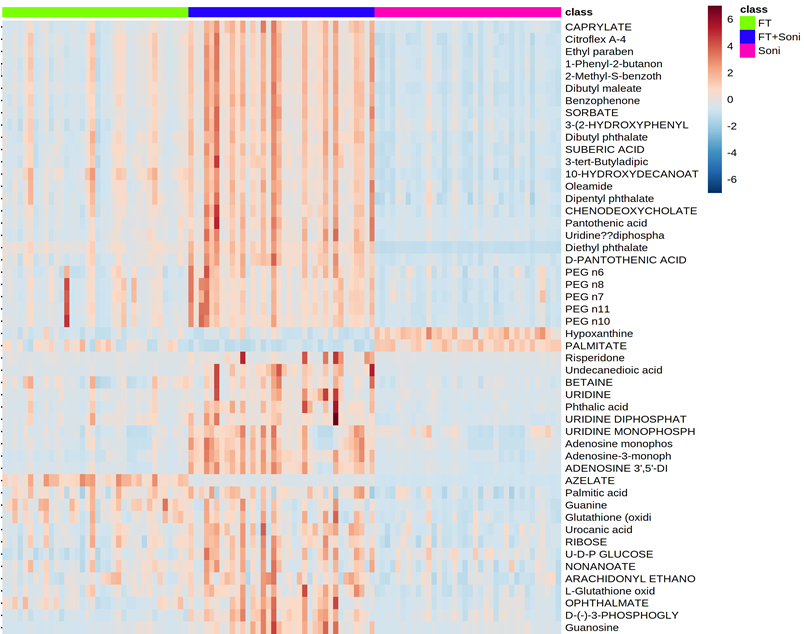


**Supplementary figure-1:** Heat map displaying qualitative expression occurred in the metabolites found common among the SC, FTC and FTC followed by SC (FTC+SC). Green = FTC, Blue=FTC+SC and Red =Sonication cycle

**Supplementary Table-1: List of other identified metabolites**

| **S.No.** | **Metabolites** | **SC** | **FTC** | **FTC+SC** |
| --- | --- | --- | --- | --- |
| 1 | 16-Hydroxyhexadecanoic acid | 3922.70 | 2641.43 | 2431.13 |
| 2 | 1-Phenyl-2-butanone | 400.45 | 401.63 | 410.58 |
| 3 | 2-Methyl-s-benzothiazole | 766.80 | 756.27 | 744.23 |
| 4 | 3-Methoxytyramine | 379.25 | 258.65 | 404.31 |
| 5 | 3-Tert-butyladipic acid | 1135.46 | 1118.25 | 1143.35 |
| 6 | 5-methylthioadenosine | 360.07 | 488.70 | 297.50 |
| 7 | Adenosine 3',5'-diphosphate | 358.17 | 313.84 | 962.14 |
| 8 | Alpha-lactose | 2331.11 | 1497.89 | 1846.38 |
| 9 | Arachidonyl ethanolamide | 1088.95 | 1170.34 | 1030.09 |
| 10 | Azelate | 563.20 | 3747.02 | 698.37 |
| 11 | Chenodeoxycholate | 853.61 | 918.52 | 961.46 |
| 12 | Citroflex a-4 | 2818.84 | 3546.86 | 3579.22 |
| 13 | D-(+)-pyroglutamic acid | 1996.87 | 1299.43 | 1430.73 |
| 14 | Dibutyl maleate | 2955.79 | 3890.09 | 4165.56 |
| 15 | Dibutyl phthalate | 457.16 | 500.09 | 509.45 |
| 16 | Diethyl phthalate | 1341.42 | 5827.34 | 6060.27 |
| 17 | Dipentyl phthalate | 4404.61 | 4692.35 | 4030.68 |
| 18 | Docosahexaenoate | 524.65 | 611.62 | 331.08 |
| 19 | Ethyl paraben | 1301.97 | 1462.72 | 1524.21 |
| 20 | Glu-Thr | 567.68 | 312.40 | 515.55 |
| 21 | Hexazinone | 548.02 | 444.25 | 624.07 |
| 22 | Methyl 2-cyano-3-(dimethylamino)acrylate | 428.42 | 367.37 | 531.99 |
| 23 | N-Acetyl leucine | 2364.73 | 1750.64 | 2717.89 |
| 24 | Ophthalmate | 327.29 | 378.20 | 357.36 |
| 25 | Palmitic acid | 134188.22 | 136607.23 | 101973.79 |
| 26 | PEG n10 | 2370.45 | 3423.54 | 3469.71 |
| 27 | PEG n11 | 1935.55 | 2772.87 | 2877.17 |
| 28 | PEG n6 | 1919.52 | 1734.83 | 1793.84 |
| 29 | PEG n7 | 2058.88 | 2318.88 | 2221.62 |
| 30 | PEG n8 | 2256.58 | 2997.16 | 2863.60 |
| 31 | Pilocarpine | 540.62 | 458.66 | 524.44 |
| 32 | Risperidone | 1303.63 | 1512.43 | 7001.70 |
| 33 | Sorbate | 332.85 | 345.94 | 396.51 |
| 34 | Suberic acid | 1136.74 | 1275.71 | 1332.17 |
| 35 | Triisopropanolamine | 1078.12 | 589.10 | 849.21 |
| 36 | Undecanedioic acid | 319.56 | 305.74 | 837.77 |
| 37 | Uridine-Diphosphate-Glucose | 6376.08 | 6524.34 | 8294.84 |
| 38 | Urocanate | 1449.88 | 808.07 | 512.13 |
| 39 | Urocanic acid | 1158.44 | 1087.99 | 1197.91 |
| 40 | 2-Hydroxyphenylacetate | - | 364.99 | - |
| 41 | 2-Octenoic acid | - | 183.92 | - |
| 42 | 3-Hydroxyphenylacetic acid | - | 381.65 | - |
| 43 | 3-Methyladenine | - | 1253.02 | - |
| 44 | 4-Hydroxycyclohexylcarboxylic acid | - | 111.10 | - |
| 45 | 4-Hydroxyestrone | - | 1137.27 | - |
| 46 | Decanoate | - | 389.19 | - |
| 47 | Diethyl 2-methyl-3-oxosuccinate | - | 1239.87 | - |
| 48 | Dipalmitoyl-phosphoethanolamine | - | 164.78 | - |
| 49 | Erucamide | - | 183247.15 | - |
| 50 | Glucose | - | 541.86 | - |
| 51 | Guanosine-3',5'-cyclic monophosphate | - | 480.28 | - |
| 52 | Hexanolactone | - | 181.98 | - |
| 53 | Hydroxyisocaproic acid | - | 667.36 | - |
| 54 | Laurate | - | 3455.03 | - |
| 55 | Lauroylcarnitine | - | 282.42 | - |
| 56 | Lyxose | - | 13773.03 | - |
| 57 | Malic acid | - | 296.78 | - |
| 58 | Mannitol | - | 6049.81 | - |
| 59 | Myristate | - | 7012.95 | - |
| 60 | Norfenefrine | - | 255.12 | - |
| 61 | O-phosphoserine | - | 855.97 | - |
| 62 | Oxaloacetic acid | - | 383.78 | - |
| 63 | PEG n13 | - | 1898.37 | - |
| 64 | Penbutolol | - | 354.37 | - |
| 65 | Phenylacetate | - | 489.03 | - |
| 66 | Phosphonoacetate | - | 1683.22 | - |
| 67 | Picolinic acid | - | 571.39 | - |
| 68 | Psicose | - | 547.44 | - |
| 69 | Ribothymidine | - | 190.41 | - |
| 70 | Sebacic acid | - | 575.56 | - |
| 71 | Uridine diphosphate-N-acetyl galactosamine | - | 470.76 | - |
| 72 | 1-Aminocyclopropanecarboxylate | - | 252.03 | 578.65 |
| 73 | 3-Alpha,11-beta,17-alpha,21-tetrahydroxy- 5-alpha-pregnan-20-one 3,21-diacetate | - | 518.53 | 1197.31 |
| 74 | 4-{[3-(diethylamino)propyl]amino}-4-oxobut-2-enoic acid | - | 364.80 | 450.49 |
| 75 | 4-Hydroxybenzaldehyde | - | 2109.79 | 2251.60 |
| 76 | 6-Hydroxycaproic acid | - | 313.64 | 408.29 |
| 77 | Benzothiazole | - | 915.19 | 828.34 |
| 78 | Dihydrosphingosine | - | 1965.27 | 568.30 |
| 79 | Elaidate | - | 30626.94 | 25840.25 |
| 80 | Ethylmethylacetic acid | - | 432.56 | 650.50 |
| 81 | Fumaric acid | - | 285.40 | 462.16 |
| 82 | Gamma-glutamylleucine | - | 671.27 | 525.92 |
| 83 | Heptanoate | - | 247.28 | 346.50 |
| 84 | Hydroxyoctanoic acid | - | 503.99 | 659.54 |
| 85 | L-(-)-carvone | - | 676.97 | 692.21 |
| 86 | Melatonin | - | 627.45 | 706.68 |
| 87 | Methohexital | - | 902.80 | 1019.78 |
| 88 | N-(4-methoxy-5-morpholino-2-nitrophenyl)-n-(2-pyridyl)amine | - | 811.26 | 1115.56 |
| 89 | Pyroglutamic acid | - | 5261.64 | 6525.21 |
| 90 | Salsolinol | - | 5.37 | 100.82 |
| 91 | 2-naphthalenesulfonic acid | - | - | 424.67 |
| 92 | 4-deoxytetronic acid | - | - | 399.06 |
| 93 | Cortisol 21-acetate | - | - | 711.84 |
| 94 | D-(+)-malic acid | - | - | 625.21 |
| 95 | Dipropyleneglycol dibenzoate | - | - | 6086.40 |
| 96 | Fipronil | - | - | 16540.92 |
| 97 | Glycerate | - | - | 265.95 |
| 98 | Leucylproline | - | - | 1032.16 |
| 99 | Melibiose | - | - | 1258.55 |
| 100 | Nicotinic acid | - | - | 508.64 |
| 101 | Oxoproline | - | - | 1816.20 |
| 102 | Phospho(enol)pyruvic acid | - | - | 741.97 |
| 103 | Stachyose | - | - | 144.65 |
| 104 | Suberate | - | - | 578.90 |
| 105 | Vanillin | - | - | 449.05 |
| 106 | 1-(carboxymethyl)cyclohexanecarboxylic acid | 428.90 | - | - |
| 107 | 13-cis-retinoic acid | 478.54 | - | - |
| 108 | 4-hydroxybenzoate | 430.71 | - | - |
| 109 | L-(+)-lactic acid | 2604.84 | - | - |
| 110 | Linoleic acid | 7463.51 | - | - |
| 111 | PEG n5 | 1702.83 | - | - |
| 112 | Taurodeoxycholic acid | 131.72 | - | - |
| 113 | Triethanolamine | 734.03 | - | - |
| 114 | N-Acetylglycine | 599.72 | - | - |
| 115 | BMPEA | 1393.81 | 1392.91 | - |
| 116 | Citrate | 614.73 | 1010.29 | - |
| 117 | Ethyl benzoate | 343.09 | 359.45 | - |
| 118 | L-glutathione (reduced) | 1606.64 | 1497.78 | - |
| 119 | N-Acetylserotonin | 1151.31 | 431.06 | - |
| 120 | PEG n12 | 1542.67 | 2317.25 | - |
| 121 | Perillartine | 1761.02 | 1561.64 | - |
| 122 | Prolylleucine | 3188.50 | 2470.42 | - |
| 123 | Trans-3-indoleacrylic acid | 644.80 | 488.93 | - |
| 124 | Uridine 5-diphospho-N-acetylglucosamine | 2084.18 | 1628.08 | - |
| 125 | Uridine 5'-diphosphate | 2942.80 | 3575.93 | - |
| 126 | Gly-leu | 2184.51 | - | 1889.52 |
| 127 | Lithocholyltaurine | 2966.08 | - | 282.50 |
| 128 | N-Acetyltryptophan | 367.55 | - | 1091.03 |
| 129 | Threonine | 477.50 | - | 332.85 |
